# 

**Published:** 1884-07

**Authors:** 


### CONTENTS.

COMMUNICATIONS.

Irregularity—A Practical Case.......... 317
Care of Children's Teeth .......... 321
How to Secure Good Dental Organs; Prevent their Decay; Prevent Rickets, Hip Diseases, etc.......... 326
Comparative Dental Anatomy.......... 328

PROCEEDINGS.

Synopsis Annual Meeting Illinois State Dental Society.......... 329
Transactions Alabama Dental Association.......... 335
Proceedings Northern Ohio Dental Society .......... 338

SELECTIONS.

Some of the Dangers and Disadvantages of Anæsthesia.......... 341
Therapeutics of Early Rising.......... 348
Nerve as a Barometer.......... 350
A Swarm of Quacks; Medical College of Ohio; Old Times.......... 351
Very Successful.......... 352

LEGAL DECISION.

Supreme Court of Illinois—Opinion.......... 352

CORRESPONDENCE.

Injurious Effects of a Rubber Plate .......... 355
On Proposed National Dental Association.......... 356
Meeting of Delegates.......... 357

SALMAGUNDI.......... 358

EDITORIAL.......... 365
American Dental Association.......... 365
National Association of Dental Examiners.......... 366
Pennsylvania State Dental Society.......... 366
Dentists' Benevolent Association.......... 366
Delegates to the American Dental Association .......... 367
Society Meetings.......... 367
Minnesota State Dental Society.......... 367
Pennsylvania State Dental Society.......... 368

